# SENIOR HOUSINGS’ ROLE FOR AGING IN PLACE: FOCUSING ON THE NATIONAL PROFILE OF INDEPENDENT LIVING FACILITIES IN KOREA

**DOI:** 10.1093/geroni/igac059.2000

**Published:** 2022-12-20

**Authors:** Sojung Park, ByeongJu Ryu

**Affiliations:** Washington University in St. Louis, St. Louis, Missouri, United States; Chuncheon Hyoja Social Welfare Center, Chuncheon-si, Kangwon-do, Republic of Korea

## Abstract

Understanding the changing preference for alternative housing arrangements is important due to the confluence of a global population aging, increasing cost concern for long-term care, and the desire of older people for aging in place (AIP). To date, little is known about to what extent the current housing models meets the needs of AIP for older people. Guided by the Person-Environment fit perspective, we examined the South Korean case using the first national data on independent living facilities (ILF) for older adults. Employing a convergent mixed-method design, we conducted a quantitative analysis of 277 ILFs and qualitative interviews with executive directors and managers from 10 facilities. We found the majority of the housings serve the low-income, and the residents' health status varies widely (i.e., ranging from fully independent to those homebound). However, the characteristics and nature of services available and staff composition are not adequate to meet the needs of the residents. Notably, privacy issues (i.e., a shared restroom and bedroom) in congregate settings stood out, decreasing the occupancy rate. Our findings suggest that the housing model needs to be diversified corresponding to the resident's needs for health and social care and economic affordability and should respect their privacy needs. We discussed ways to reform the current policy governing the ILFs, including strengthening service coordination programs to alleviate gaps between the residents' needs and the service availability. Our findings provide implications for improving and reforming the existing housing model to age in place in Korea and potentially other aging societies.

